# Philo-Medicus, on a General List of Practitioners

**Published:** 1801-11

**Authors:** 

**Affiliations:** Scotland


### *To the Editors of the Medical and Phyſical Journal.*

GENTLEMEN,

IN Number XXXII. of your uſeful Journal, I addreſſed you on the importance of publiſhing in ſome reſpectable almanack, a correct liſt of all the medical practitioners in the kingdom. I am happy to ſee that it has had ſome effect, and hope that every perſon whoſe intereſt it concerns, will cheerfully come forward and ſupport the cauſe. It is with no ſmall degree of pleaſure I obſerved that the Editors of the Britiſh Almanack, publiſhed in Edinburgh and Glaſgow, have taken up the ſubject, and printed in a number of newſpapers, the following advertiſement.

"To the Phyſicians and Surgeons in Scotland.

"The Publiſhers of the Britiſh Almanack, at the requeſt of ſeveral gentlemen of the Faculty, propoſe to publiſh in their almanack for 1802, a liſt of all the practiſing Phyſicians and Surgeons in Scotland, who have obtained regular Degrees and Diplomas. It is therefore requeſted, that thoſe concerned will furniſh them with their names, place of reſidence, the date of their degree or diploma, and the faculty from which it was obtained.

"Letters may be addreſſed to Alexander Chapman and Co. printers, Forreſter's Wynd, Edinburgh. None will be attended to, unleſs poſt paid."

The publiſhers of the Britiſh Almanack have thought it unneceſſary to require of every practitioner a formal certificate of his *Degree* or *Diploma*, truſting that one practitioner will be a check upon another; and ſhould any have the temerity to impoſe upon them, and be detected, their names ſhall be expoſed in n the moſt public manner, to prevent the like fraud in others They ſhall be held forth as QUACKS and IMPOSTORS to the whole world. I am, &c.

*Scotland, Oct.* 12, 1801. PHILO-MEDICUS.

P. S. Your Journal is now ſo *univerſally* read, that there is ſcarcely a practitioner in Scotland without a copy of it; I have therefore choſen this method of making the advertiſement *univerſally* known.

